# Marfan syndrome with a complex chromosomal rearrangement including deletion of the *FBN1 *gene

**DOI:** 10.1186/1755-8166-5-5

**Published:** 2012-01-19

**Authors:** Mileny ES Colovati, Luciana RJ da Silva, Sylvia S Takeno, Tatiane I Mancini, Ana R N Dutra, Roberta S Guilherme, Cláudia B de Mello, Maria I Melaragno, Ana B A Perez

**Affiliations:** 1 Departamento de Morfologia e Genética, Universidade Federal de São Paulo, São Paulo, SP, Brasil; 2 Departamento de Psicobiologia, Centro Paulista de Neuropsicologia, Universidade Federal de São Paulo, São Paulo, SP, Brasil

**Keywords:** *FBN1*, Marfan syndrome, Complex Chromosomal Rearrangement

## Abstract

**Background:**

The majority of Marfan syndrome (MFS) cases is caused by mutations in the fibrillin-1 gene (*FBN1*), mapped to chromosome 15q21.1. Only few reports on deletions including the whole *FBN1 *gene, detected by molecular cytogenetic techniques, were found in literature.

**Results:**

We report here on a female patient with clinical symptoms of the MFS spectrum plus craniostenosis, hypothyroidism and intellectual deficiency who presents a 1.9 Mb deletion, including the *FBN1 *gene and a complex rearrangement with eight breakpoints involving chromosomes 6, 12 and 15.

**Discussion:**

This is the first report of MFS with a complex chromosome rearrangement involving a deletion of *FBN1 *and contiguous genes. In addition to the typical clinical findings of the Marfan syndrome due to *FBN1 *gene haploinsufficiency, the patient presents features which may be due to the other gene deletions and possibly to the complex chromosome rearrangement.

## Background

Marfan syndrome (MFS) is a dominant disorder, mainly caused by mutations in the fibrillin-1 gene (*FBN1*) located on chromosome 15q21.1. The estimated prevalence of MFS is about 1 in 10000. Approximately 25% of MFS patients are sporadic cases due to new mutations [1,2]. Different tissues and organs can be affected, especially the cardiovascular, skeletal, and ocular systems. Diagnostic criteria are well established and known as the Ghent criteria [3]. However, the inter- and intra-familial variability of the phenotype limits the establishment of genotype-phenotype correlations. To date, more than 1329 *FBN1 *mutations have been published (http://www.hgmd.cf.ac.uk/ac/gene.php?gene=FBN1), but only a few are recurring mutations. Missense mutations substituting or creating a cysteine molecule in one of the calcium-binding EGF domains are the most prevalent. The others are frameshift, splice-site, nonsense mutations and in-frame deletions and insertions. Heterozygous mutations in the genes coding for transforming growth factor beta receptors I (*TGFBR1*) and II (*TGFBR2*) have also been reported in patients with MFS and MFS-related disorders, indicating genetic heterogeneity [4-6]. Interstitial deletions involving the 15q21.1 band and the *FBN1 *gene are very rare. To our best knowledge, there are only six reports in the literature describing deletions of the whole *FBN1 *gene detected by molecular techniques, and only in five of them this gene deletion was associated with classical MFS [2,7-11]. The patients described by Adès et al. [8] and Hutchinson et al. [7] had clinical features of the MFS spectrum and mental retardation, but the size of their deletions was not determined. The patient described by Faivre et al. [10] had a 2.97 Mb deletion and some features of MFS but no mental retardation. Hilhorst-Hofstee et al. [2] described 10 cases (five of the same family) with a complete *FBN1 *gene deletion, screened by Multiplex Ligation-dependent Probe Amplification (MLPA) analysis of 300 patients presenting from mild MFS features to the classical MFS or an MFS-related phenotype. Recently, Furtado et al. [11] studied 14 patients from 11 unrelated families with aortic aneurysm. Three patients of the one family who met clinical diagnostic criteria for Marfan syndrome had a 542 Kb deletion in chromosome 15 including the whole *FBN1 *gene; the study of the region was refined by MLPA and array analysis. Cases with chromosomal alterations including a genomic deletion of the whole *FBN1 *gene are rare, but their number has increased after the adoption of routine screening by molecular techniques (MLPA, *array*), especially in patients with MFS. To date, there is no description in the literature of a patient with MFS and a complex chromosome rearrangement (CCR). We report here on a 16-year-old female patient displaying features of the MFS spectrum and mental retardation, who was found to present a 1.9 Mb deletion at 15q21.1 (refined by array) including the *FBN1 *gene, and a novel CCR among chromosomes 6, 12 and 15.

## Case presentation

**Figure 1:**
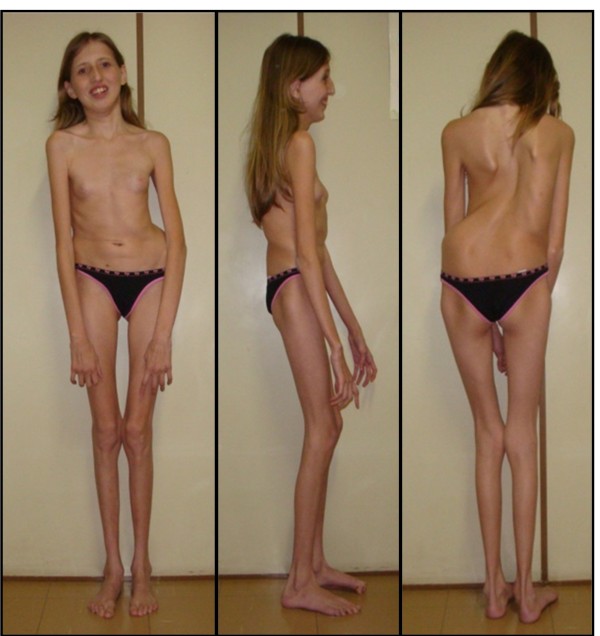
**Patient at age 13 years**.

The proband (Figure 1) is the first child of healthy parents. She was born at term, with a length of 50 cm and a weight of 3240 g. She walked at 18 months, and spoke at four years of age. She was however able to attend a regular school, but showed hyperactivity and difficulty to focus. She had a seizure at the age of 3 years, and a cardiac examination evidenced mitral insufficiency with a dystrophic valve. An evolutive scoliosis was noted at the age of 6 years, with progression. At age 11, hypothyroidism was detected. She was referred at 13 years of age for a suspicion of MFS, showing: positive thumb and wrist sign, scoliosis, joint hyperlaxity, high-arched palate with dental crowding, dysmorphism and aortic root dilatation with dystrophic mitral valve. Ophthalmological examination revealed myopia and astigmatism but not ectopia lentis. A neuropsychological assessment showed global intellectual impairment (IQ 50) according to the Brazilian values of the Wechsler Intelligence Scale for Children-III (WISC-III), with major deficits in attention and executive skills. Thus, she met the Ghent criteria for Marfan syndrome, but also presented craniostenosis, hypothyroidism and intellectual deficiency.

### Genetic Study

#### Classical Cytogenetic Findings

**Figure 2:**
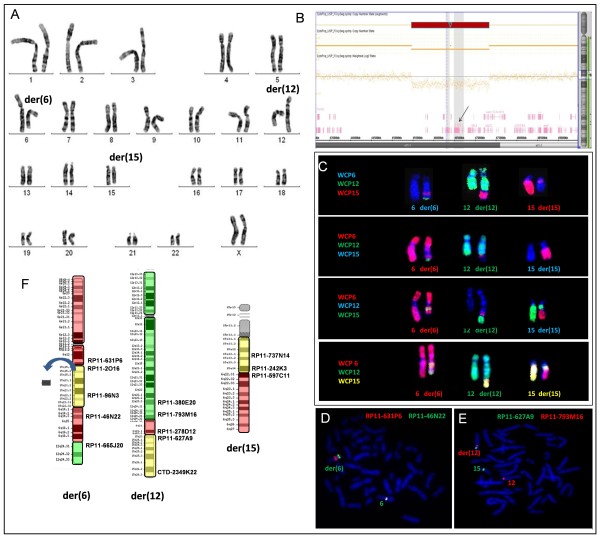
**Cytogenetic and molecular data from the patient studied**. A) GTG-banded chromosomes showing the translocation involving chromosomes 6, 12 and 15. B) Array result for chromosome 15 showing the 1.9 Mb deletion (red) at 15q21.1 including the *FBN1 *gene (arrow). C) FISH with WCP probes of chromosomes 6, 12 and 15 in different color combinations showing a complex chromosomal rearrangement. D) FISH with probes RP11-631P6 (6q13) in red and RP11-46N22 (6q14.3) in green, showing signals next to each other on the normal chromosome 6 and separate signals on the der (6) chromosome. E) FISH with probes RPRP11-627A9 (15q23) in green and RP11-793M16 (12q24.13) in red, showing signals of both on the derivative chromosome 12. F) Ideogram of the derivative chromosomes involved in the patient's complex chromosome rearrangement, showing the probes used to define the breakpoints and the 15q21.1 band deletion (arrow).

Conventional chromosome analysis was performed on phytohemagglutinin-stimulated lymphocytes from peripheral blood cultures, using GTG banding according to standard protocols. Cell images were captured using the Ikaros Digital Imaging System (Metasystem, Altlussheim, Germany). G-banded analysis with a resolution of 550 bands per haploid karyotype revealed a female karyotype with a *de novo *balanced translocation involving chromosomes 6, 12 and 15, with breakpoints apparently at 6q22, 12q24 and 15q21 (Figure 2A).

#### Molecular Findings

Genomic DNA was isolated from peripheral blood using a Gentra Puregene kit (Qiagen Sciences, Inc., Germantown, MD). Array study was performed with Affymetrix Cytogenetics Array 2.7 (Affymetrix Inc., Santa Clara, CA, USA) according to the manufacturer's instructions, using the Affymetrix Chromosome Analysis Suite software. Copy number state indicated an apparently contiguous interstitial deletion (~1.9 Mb) on chromosome 15q21.1 in the region 45,466,733-47,335,104 bp (NCBI36/hg18) (Figure 2B). Parental array analysis showed normal results.

#### Molecular Cytogenetic Findings

In order to better characterize the complex chromosome rearrangement, FISH was performed with region-specific BAC probes for chromosomes 6q, 12q and 15q. Clones were selected from the BACPAC Resource Center at the Children's Hospital Oakland Research Institute (Oakland, CA, USA) and prepared according to Guilherme et al. [12]. FISH was performed with whole chromosome painting (WCP) probes for chromosomes 6, 12 and 15 (Cytocell, Cambrigde, UK). Individual and combined WCP probes were used in order to clarify the complex chromosomal rearrangement. Cell images were captured using the Isis Digital Imaging System (Metasystem, Altlussheim, Germany). FISH analysis revealed a quite complex rearrangement with eight breakpoints, as follows:

46, XX, t(6;12;15)(6pter→6q14::15q15.1→15q21.1::15q21.1→15q22.3::6q14→6q21::12q24.1→12qter;12pter→12q24.1::6q21→6q22.2::15q22.3→15qter;15pter→15q15.1::6q22.2→6qter)dn.arr 15q21.1(45,466,733-47,335,104)×1 (Figure 2C, D and 2E).

## Discussion

We report here on a girl with clinical features of the MFS spectrum and a 15q21.1 deletion including the entire *FBN1 *gene. This is the seventh study in the literature in which the deletion of *FBN1 *is confirmed by molecular techniques. Thus, to this date 18 patients with deletions including the whole *FBN1 *gene were reported, five of them belonging to the same family. Interestingly, only 12 of these patients [2,7,11, present case] present the typical MFS phenotype according to the Ghent criteria. Clinical variability in patients with different point mutations has been described, with patients with a nonsense mutation presenting milder phenotypes than patients with a missense mutation [10]. Cases with a deletion can be compared to patients with a nonsense mutation, since the truncated mRNA in the latter cases is believed to be reduced, due to the nonsense-mediated decay (NMD) mechanism that prevents the expression of the truncated mRNA [2,10,13]. Thus, in cases of deletion and nonsense mutation, the MFS phenotype can result from *FBN1 *gene haploinsufficiency and, especially in cases of missense mutations, from a dominant negative effect. Hutchinson et al [7] suggested that the clinical variability in MFS could be also due to variable *FBN1 *expression of the normal allele. The size of the deletions reported varies from small (less than 300 kb in 5 members of the same family) [2], including only the *FBN1 *gene, to large (up to 17.7 Mb) [9]. As found in our patient, when the deletion involves other genes besides *FBN1*, other unusual features can be found, such as those described here (craniostenosis, hypothyroidism and intellectual deficit). The patient described by Hiraki et al [9] presented no sign of the MFS syndrome, probably due to her young age and severe clinical phenotype. Our patient showed most of the skeletal features of MFS and an aortic root dilatation, but no ectopia lentis. Concerning the ocular system, she presented myopia and astigmatism. Her deletion comprises 19 genes and predicted genes including *FBN1*, besides *SEMA6D *and *COPS2 *that may have contributed to the intellectual deficit and hypothyroidism, respectively. Of the 18 patients with a complete deletion of the *FBN1 *gene described so far, only seven were karyotyped: two presented normal karyotypes [2,10], three had visible 15q deletions [7-9], and one had a de novo translocation between the long arms of chromosomes 12 and 15 and a 4.9 Mb interstitial deletion at the translocation breakpoint of the long arm of chromosome 15 between the bands q21.1 and q21.2 [2]. Hilhorst-Hofstee et al [2] performed karyotype analysis only in two patients, as part of the mental retardation screening. So, our patient is the first MFS case described presenting a complex chromosome rearrangement among chromosomes 6, 12 and 15. In the literature, up to 30-50% of the patients with a chromosomal rearrangement, both complex and reciprocal translocations, show an imbalance on the chromosomal or molecular level as an explanation for their phenotype [14]. The greater the number of breakpoints involved in a CCR, the greater the likelihood of genomic imbalances or position effect. Disruption of a gene could unmask a recessive mutation on the homologue allele, suggesting a greater chance for an abnormal phenotypic outcome [15].

## Conclusion

We emphasize the importance of using a combination of different molecular cytogenetic techniques in cases of chromosomal and/or genomic rearrangements involving the *FBN1 *gene, in order to better understand the extent of the molecular etiology of the Marfan syndrome and also to elucidate the genetic constitution of CCRs associated with diseases.

### Consent

Written informed consent was obtained from the patient's parents for the publication of this case report and accompanying images. A copy of the consent form is available for review by the Editor-in-Chief of this journal.

## Competing interests

The authors declare that they have no competing interests.

## Authors' contributions

MESC performed the molecular karyotyping and data analysis and wrote the manuscript; ABAP made the clinical evaluation of the patient; SST, TIM, ARND and RSG did the molecular analysis (FISH, WCP and SNP array); CBM performed a neuropsychological evaluation of the patient; LRJS made the cytogenetic analysis; and ABAP and MIM coordinated the study. All the authors have read and approved the manuscript.
